# Supplementary figures and images for: Endocannabinoid signaling is a critical link between circadian desynchronization and metabolic dysfunction
Source: bioRxiv. 2025 Oct 1:2025.09.29.678590. Preprint. [Version 1] doi: 10.1101/2025.09.29.678590 (PMC12621973; doi:10.1101/2025.09.29.678590)

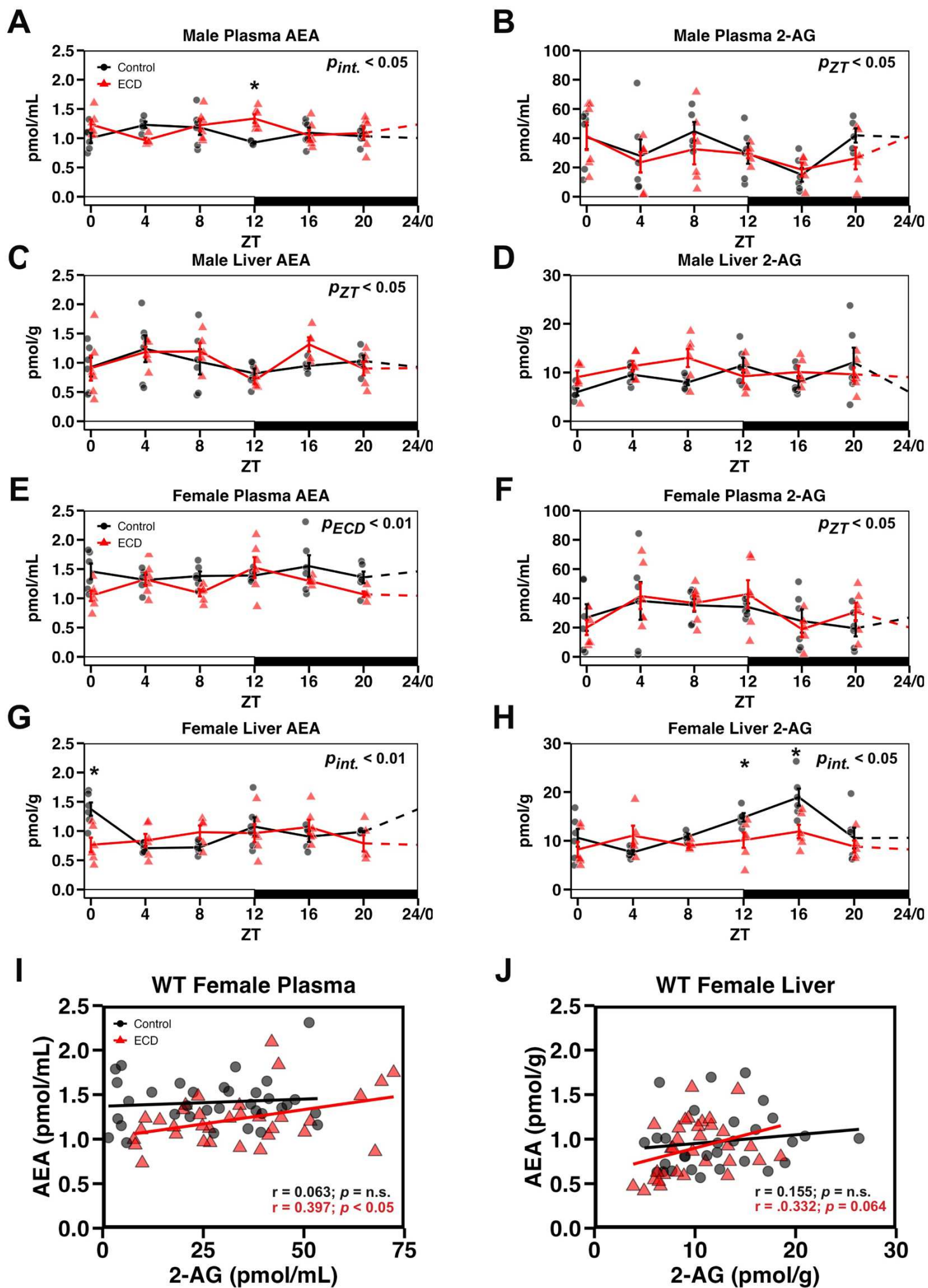

A

Female Weight Gain

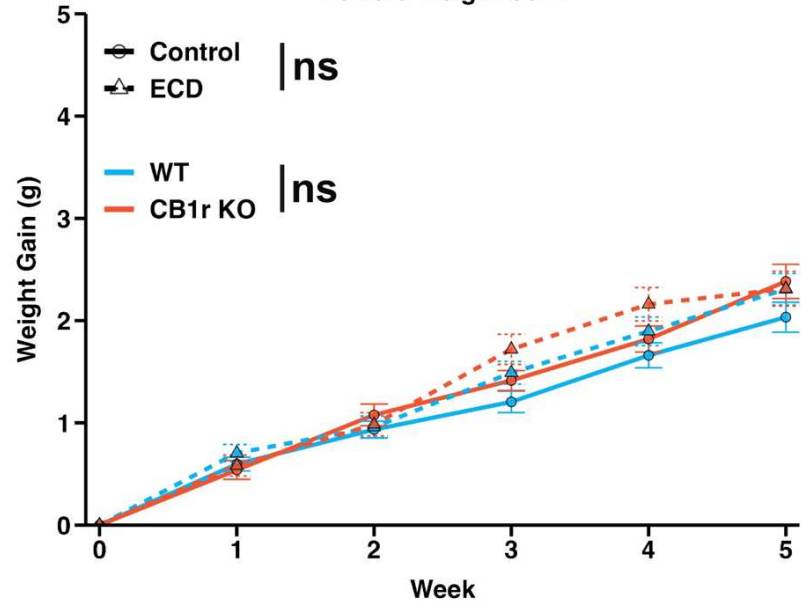

**A**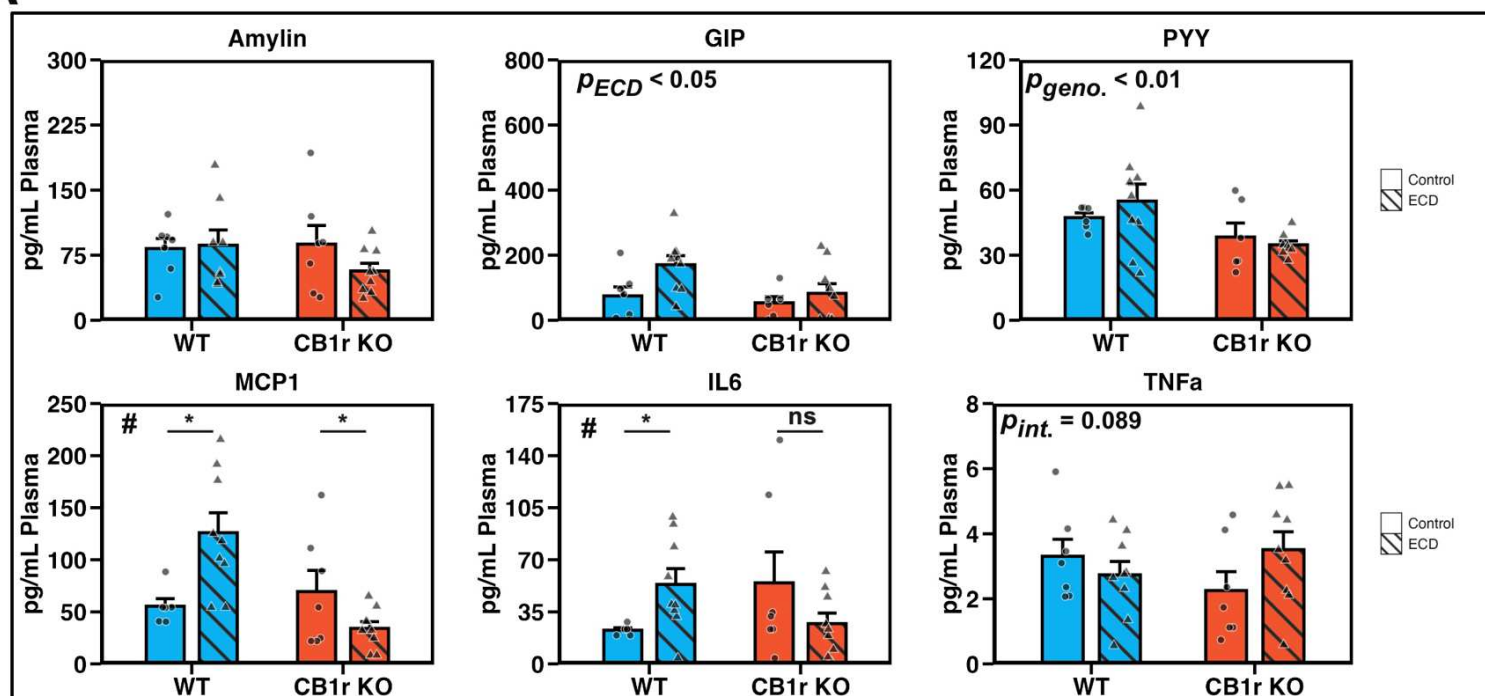**B**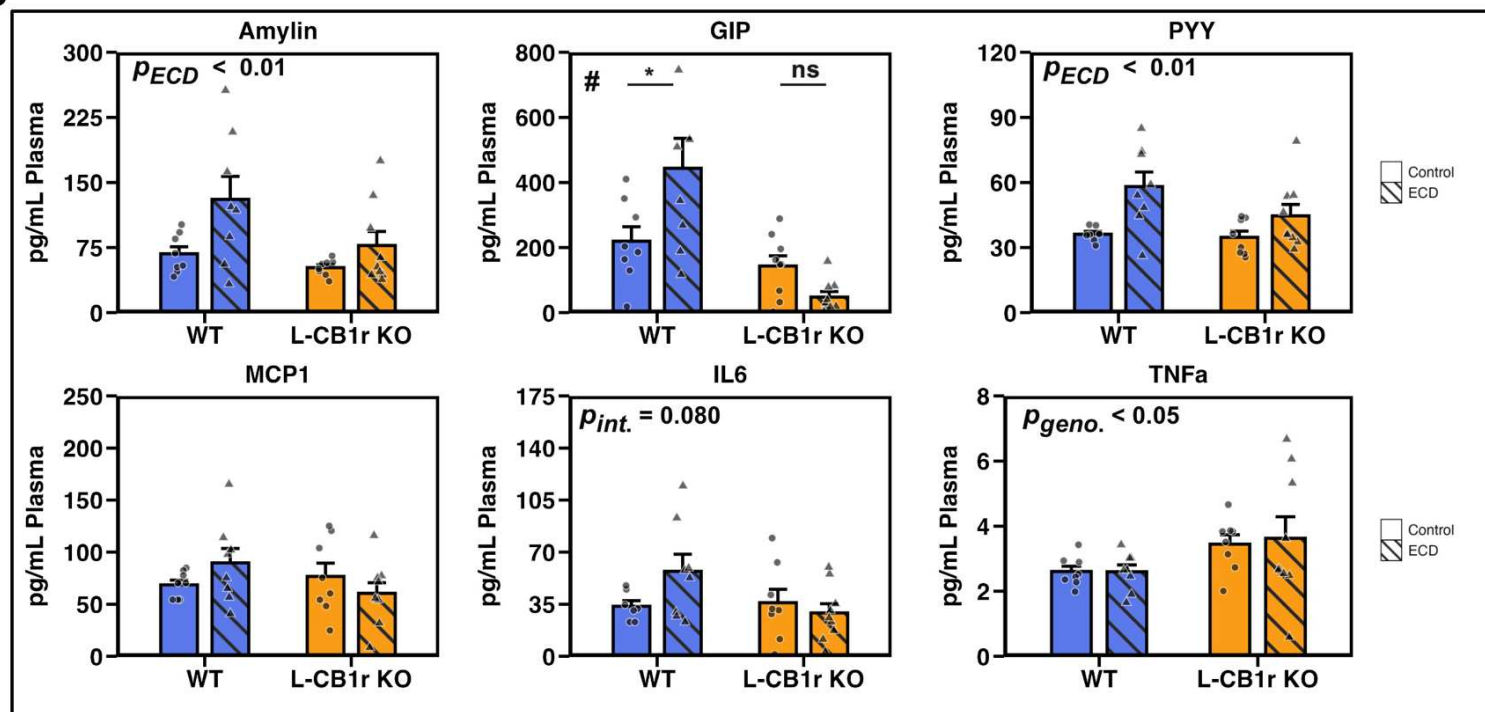**C**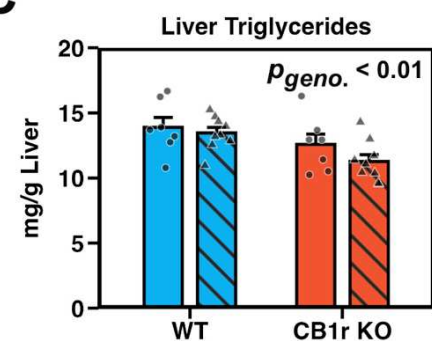**D**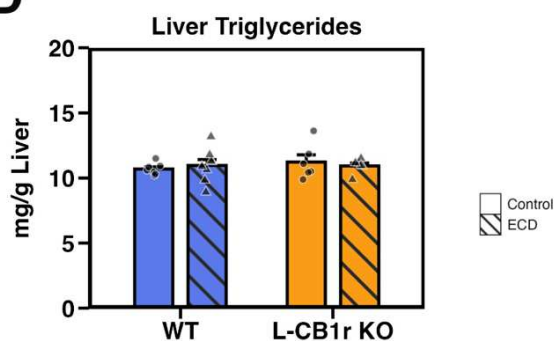

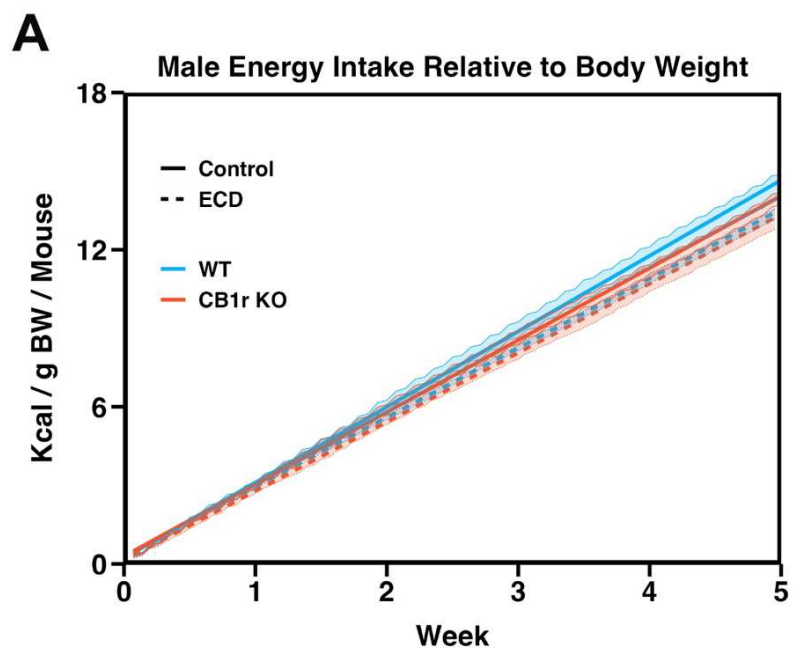

A

Locomotion

Feeding

WT  
Control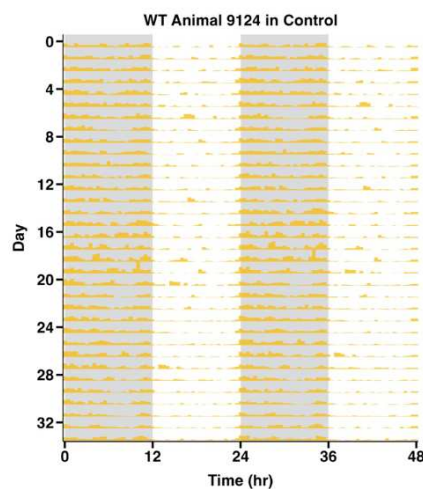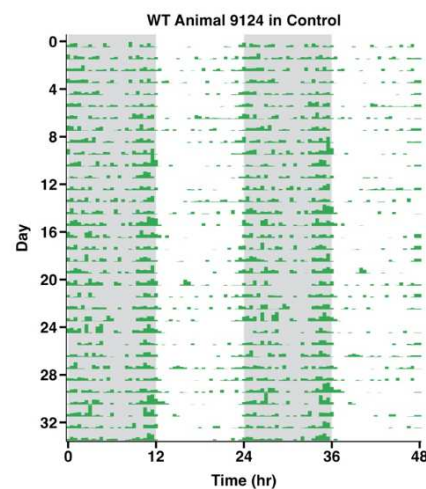CB1r KO  
Control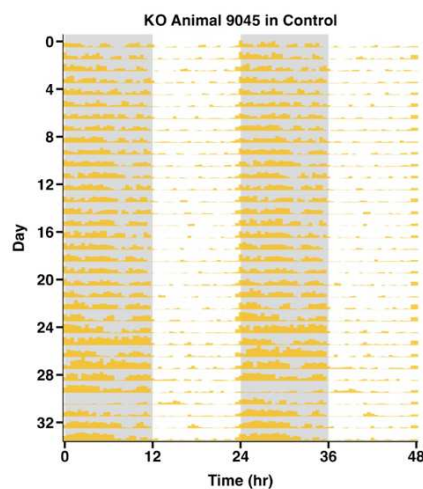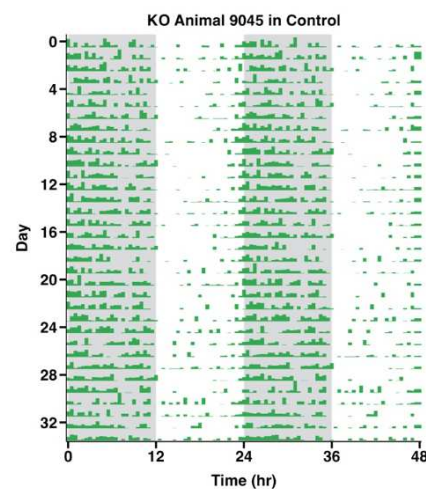WT  
ECD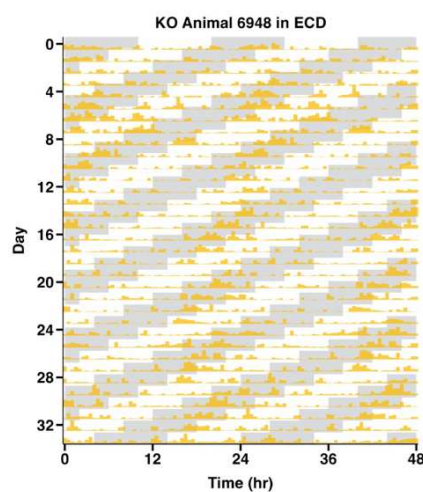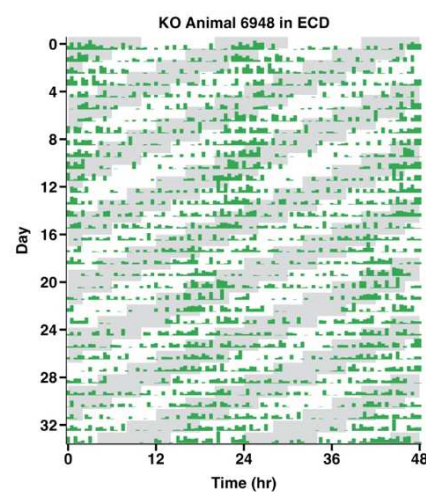CB1r KO  
ECD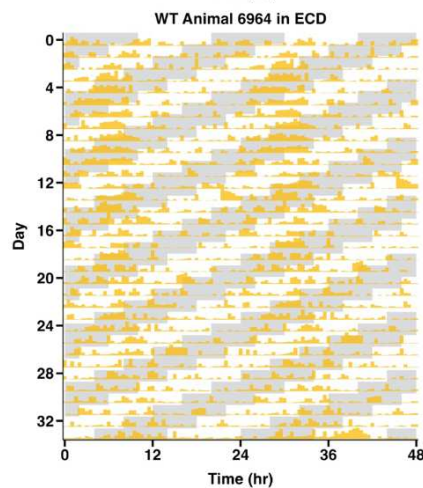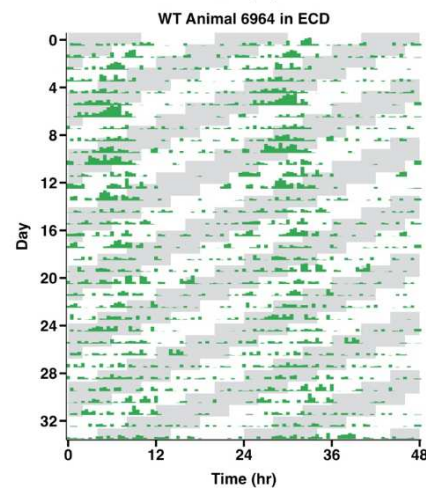

A

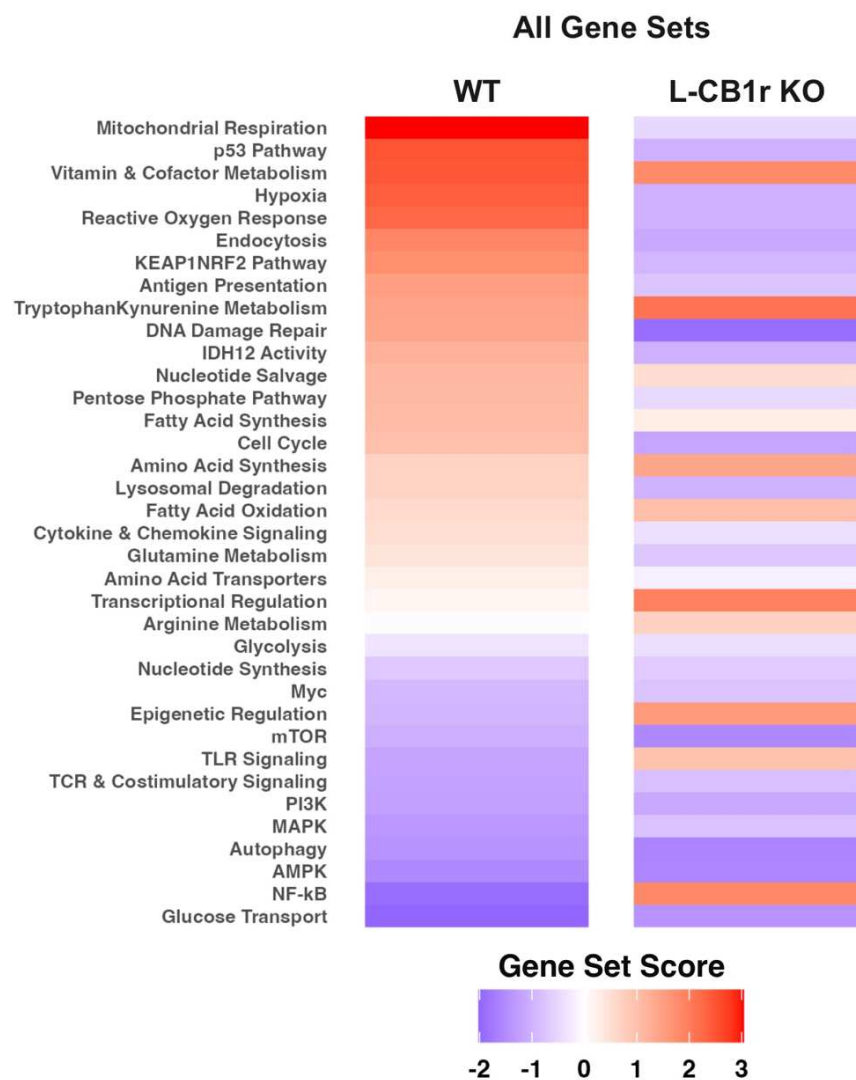

Supplement: Supplement 1 [file media-1.pdf]
